# Supplementary material for: Effectiveness of Transition Care Intervention Targeted to High-Risk Patients to Reduce Readmissions: Study Protocol for the TARGET-READ Multicenter Randomized-Controlled Trial
Source: Healthcare (Basel). 2023 Mar 18;11(6):886. doi: 10.3390/healthcare11060886 (PMC10048511; doi:10.3390/healthcare11060886)
Supplement: Supplementary file 1 [file healthcare-11-00886-s001.zip › healthcare-2273552_supplementarymaterial_information_atrialfibrillation_patient.pdf]

[Logo of the recruiting center]

Information sheet to the patient
ATRIAL FIBRILLATION

What is atrial fibrillation?
Atrial fibrillation is the most common heart rhythm disorder. It is characterized by an irregular and often too rapid rhythm that prevents adequate blood flow. This can lead to the formation of blood clots in the heart with a risk that these clots will travel to the brain and cause a stroke. Fibrillation can be manifested by palpitations (heartbeat), malaise, heart failure. The most feared complication is stroke.

What are the goals of treatment?
To slow down the heart rate when it is too fast or to bring the heart rate back to a normal rhythm when possible. The goal is also to reduce the risk of complications such as stroke.

What is the treatment?
1) Medication to slow the heart rate or maintain a normal rhythm.
2) Medication to "thin" the blood, and thus reduce the risk of stroke.
3) Sometimes restoring normal heart rhythm via an electric shock (cardioversion), or cardiac intervention to cut the defective electrical circuit (ablation).

When should I see a doctor?
Contact your doctor when you experience any of the following symptoms:
- feelings of discomfort, palpitations (heartbeat), unusual fatigue, the onset of greater difficulty in breathing.

Contact 144 if you experience:
- Sudden difficulty breathing.
- Chest pain.
- Loss of consciousness.
- Symptoms suggestive of a stroke: paralysis of part of the face, difficulty speaking, weakness of an arm or leg.

What should I watch out for?

Measure my pulse regularly, and if it is too fast or too slow, consult my doctor. The normal heart rate is between 60 and 100 beats per minute, ideally between 60 and 80.

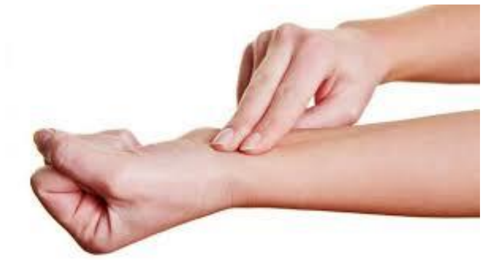

What can I do for my health if I have atrial fibrillation?

1) Consult my GP immediately if new symptoms appear.
2) Take my medications as prescribed and go to my GP for check-ups according to his recommendations.
3) Know how to measure my pulse.
