# Supplementary material for: Effectiveness of Transition Care Intervention Targeted to High-Risk Patients to Reduce Readmissions: Study Protocol for the TARGET-READ Multicenter Randomized-Controlled Trial
Source: Healthcare (Basel). 2023 Mar 18;11(6):886. doi: 10.3390/healthcare11060886 (PMC10048511; doi:10.3390/healthcare11060886)
Supplement: Supplementary file 1 [file healthcare-11-00886-s001.zip › healthcare-2273552_supplementarymaterial_information_chronicrenalfailure_patient.pdf]

[Logo of the recruiting center]

Information sheet to the patient
CHRONIC RENAL FAILURE

What is chronic kidney disease?
The kidneys act as a sort of purification station for the blood. They also play a major role in controlling blood pressure and forming red blood cells. Kidney failure occurs when the kidneys are no longer able to do their job. High blood pressure and diabetes are common causes of kidney failure. Kidney failure can be manifested by, for example, water accumulation (edema), fatigue, anemia.

What are the goals of treatment?
To slow down the progression of renal failure and therefore the risk of complications. To delay the eventual need for kidney replacement by dialysis.

What is the treatment?
1) A combination of medications to treat cardiovascular risk factors and thus slow down the progression of the disease.
2) Vaccination against the flu every year end.
3) In case of advanced disease: dialysis or even kidney transplantation.

When should I see a doctor?
Contact your doctor when you have any of the following symptoms:
- Swollen legs, weight gain, increased fatigue, lack of appetite, nausea or vomiting, dehydration, decreased urine output.

Contact 144 if you experience:
- Sudden difficulty breathing.
- Chest pain.
- Faintness or loss of consciousness.

[Logo of the recruiting center]

What can I do for my health if I have chronic kidney failure?

1) See my doctor regularly, or if not, my nephrologist.
2) Take my medications as prescribed. In case of problems with medication, do not stop taking it without contacting my doctor or pharmacist promptly.
3) Avoid taking medications without my doctor's approval (e.g. anti-inflammatory drugs).
4) Weigh myself regularly, keep track of my weight and consult my GP if I gain weight.
5) Avoid dehydration: drink between 1 and 1.5L/day, unless contraindicated by my doctor.
6) Get a flu shot every year.
7) Avoid eating too many foods rich in potassium (bananas, dried fruits).
