# Supplementary material for: Effectiveness of Transition Care Intervention Targeted to High-Risk Patients to Reduce Readmissions: Study Protocol for the TARGET-READ Multicenter Randomized-Controlled Trial
Source: Healthcare (Basel). 2023 Mar 18;11(6):886. doi: 10.3390/healthcare11060886 (PMC10048511; doi:10.3390/healthcare11060886)

[Logo of the recruiting center]

Target READ Trial — FUNDED BY THE SWISS NATIONAL SCIENCE FOUNDATION

Information sheet to the patient
LIVER CIRRHOSIS

What is cirrhosis of the liver?
The liver is an essential organ with multiple functions such as detoxification of blood or production of proteins. Cirrhosis is a condition that is usually irreversible due to damage to the liver, most often caused by toxic substances (alcohol) or infection. Symptoms of cirrhosis are most often fatigue, lack of appetite, fluid accumulation in the stomach and legs, jaundice. Cirrhosis increases the risk of digestive bleeding and liver cancer.

What are the goals of treatment?
To slow down the progression of the disease and avoid complications. In order to do this, it is essential to avoid any further damage to the liver.

What does the treatment usually consist of?
1) Treat the cause.
2) Stopping the consumption of all substances that are toxic for the liver: alcohol, various medications as recommended by my doctor.
3) Up-to-date vaccinations, especially against flu and hepatitis.
4) Avoid eating a diet too rich in salt in case of liquid in the stomach.
5) Make sure you have a regular bowel movement every day, take laxatives if necessary.
6) Take my medications as prescribed.
7) Abdominal punctures to remove excess fluid are sometimes necessary.

When should I see a doctor?
Contact your doctor when you have any of the following symptoms:
- Swollen legs, weight gain, increased difficulty breathing, or fatigue on exertion, lack of appetite, fever, confusion.

Contact 144 in case of:
- Vomiting blood or very black vomit.
- Fainting or loss of consciousness.

What can I do for my health if I have cirrhosis of the liver?

1) Take my medications regularly and avoid taking any medications without talking to my doctor.
2) Visit my doctor regularly.
3) Check my weight and waistline regularly.
4) Get a flu shot every year.
5) Stop smoking, have a healthy lifestyle, avoid eating salty meals.

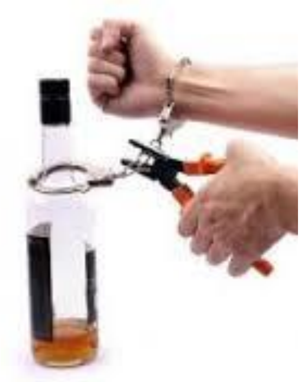

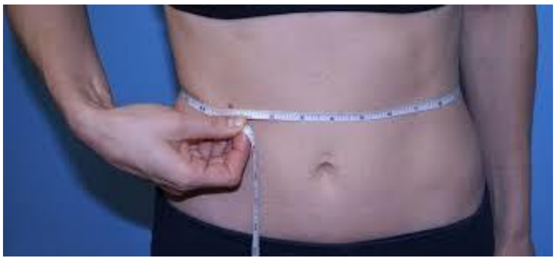

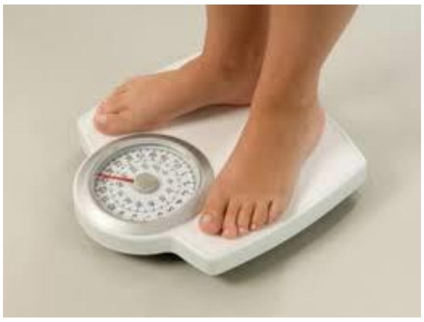

Supplement: Supplementary file 1 [file healthcare-11-00886-s001.zip › healthcare-2273552_supplementarymaterial_information_cirrhosis_patient.pdf]
