# Supplementary material for: Effectiveness of Transition Care Intervention Targeted to High-Risk Patients to Reduce Readmissions: Study Protocol for the TARGET-READ Multicenter Randomized-Controlled Trial
Source: Healthcare (Basel). 2023 Mar 18;11(6):886. doi: 10.3390/healthcare11060886 (PMC10048511; doi:10.3390/healthcare11060886)
Supplement: Supplementary file 1 [file healthcare-11-00886-s001.zip › healthcare-2273552_supplementarymaterial_information_controlgroup_patient.pdf]

[Logo of the recruiting center]

Information sheet – control group

Participation at the TARGET-READ study

Madame, Sir,

Thank you for participating in the Target-READ study, which focuses on reducing the risk of rehospitalization for patients.

You have been selected to be part of the control group, and as such you will benefit from a normal discharge from the hospital. Your family doctor will soon receive a letter from the hospital with information about your hospital stay.

As announced, **someone will contact you by phone within a month** to ask you some questions, especially if you had to return to the hospital.

We thank you in advance for your very valuable cooperation.

The entire study team wishes you a safe return home and a speedy recovery.

For the research team, Professor Jacques Donzé

Contact in case of questions about the study:

**Study nurse**	**Physician locally responsible for the study at your hospital**	**Physician responsible for the study**
[name]<br>[address]<br>[email]<br>[phone number]	[name]<br>[address]<br>[email]<br>[phone number]	Prof. Dr. med. Jacques Donzé<br>[address]<br>[email]<br>[phone number]
