# Supplementary material for: Effectiveness of Transition Care Intervention Targeted to High-Risk Patients to Reduce Readmissions: Study Protocol for the TARGET-READ Multicenter Randomized-Controlled Trial
Source: Healthcare (Basel). 2023 Mar 18;11(6):886. doi: 10.3390/healthcare11060886 (PMC10048511; doi:10.3390/healthcare11060886)

[Logo of the recruiting center]

Information sheet to the patient

CHRONIC OBSTRUCTIVE PULMONARY DISEASE

What is chronic obstructive pulmonary disease (COPD)?
COPD is a chronic inflammation of the bronchial tubes associated with lung destruction, most often caused by smoking. The direct consequences are difficulty breathing, with the risk of acute exacerbations and/or respiratory infections becoming more frequent.

What is an exacerbation and how to recognize it?
It is an acute phase of deterioration, with: increased difficulty breathing, coughing and/or sputum, sometimes associated with a change in sputum color or fever.

What are the goals of treatment?
To improve shortness of breath, to reduce the risk of acute exacerbations and therefore hospitalizations, and to slow down the progression of the disease.

What does the treatment usually consist of?
1) Stop smoking: this is the only way to slow down the disease!
2) Vaccination against the flu at the end of each year, and against pneumococcus.
3) Inhaled medication (bronchodilators).
4) During acute exacerbations: cortisone tablets and antibiotics.
5) Rehabilitation program with breathing techniques.
6) In more severe cases, home oxygen or surgery.

When should I see a doctor?
When I have a worsening or the appearance of any of the following symptoms:
- Cough.
- Sputum that is larger or changes color.
- Increased difficulty breathing.
- Fever or flu-like symptoms.

Contact 144 if you have
- Severe or sudden difficulty breathing.
- Chest pain.
- Faintness or loss of consciousness.

What can I do for my health if I have COPD?

1) Take my medication regularly and according to the correct inhalation technique. If I am unsure, ask my doctor.
2) Get a flu shot every year.
3) Stop smoking and get help from my doctor.
4) Hygiene measures, especially in winter: wash your hands regularly, especially after being in public places.
5) Visit my GP regularly for check-ups, and as soon as symptoms of an exacerbation appear.
6) Stay active as much as possible.

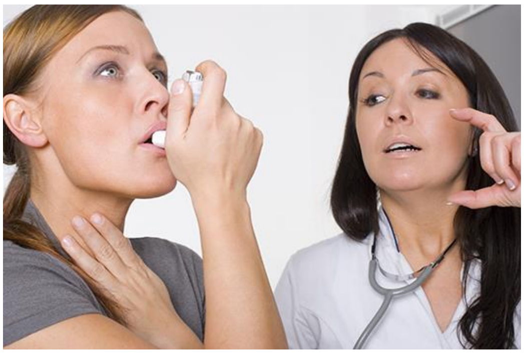

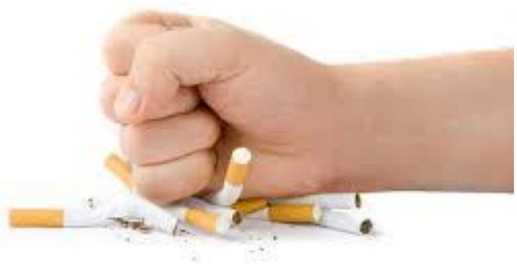

Supplement: Supplementary file 1 [file healthcare-11-00886-s001.zip › healthcare-2273552_supplementarymaterial_information_copd_patient.pdf]
