# Supplementary material for: Effectiveness of Transition Care Intervention Targeted to High-Risk Patients to Reduce Readmissions: Study Protocol for the TARGET-READ Multicenter Randomized-Controlled Trial
Source: Healthcare (Basel). 2023 Mar 18;11(6):886. doi: 10.3390/healthcare11060886 (PMC10048511; doi:10.3390/healthcare11060886)

[Logo of the recruiting center]

Information sheet to the patient
CORONARY DISEASE / INFARCTION

What is coronary heart disease?
It is the suffering or death of a part of the heart caused by the lack of oxygen supply, most often due to the obstruction of a vessel of the heart (the coronary arteries). This is manifested by chest pain that can occur during exercise, or in the case of more severe damage even at rest. When an artery is completely blocked, it causes a heart attack (infarction).

What are the goals of treatment?
The main goals of treatment are to reduce symptoms, slow the progression of the disease, reduce the risk of heart attack and death, and improve quality of life.

What does the treatment usually consist of?
1) Medication to reduce the risk of coronary obstruction (Aspirin, Plavix).
2) Medication to reduce blood pressure and protect the heart.
3) Medication to treat other diseases that can lead to coronary heart disease, such as diabetes or high cholesterol.

When should I see a doctor?
Contact your doctor if you experience increased chest discomfort or new chest pain.

Contact 144 if you experience
- oppressive chest pain (sometimes radiating to the jaw, left arm or back).
- difficulty breathing.

[Logo of the recruiting center]

What can I do for my health if I have coronary heart disease?

1) See my doctor regularly, but especially when I notice that I sometimes have chest pains.
2) Take my medication as prescribed. In case of problems related to medication, do not stop taking it without contacting my doctor or pharmacist promptly.
3) Have a healthy lifestyle: stop smoking, limit fatty foods, have regular physical activity (see general information sheet).

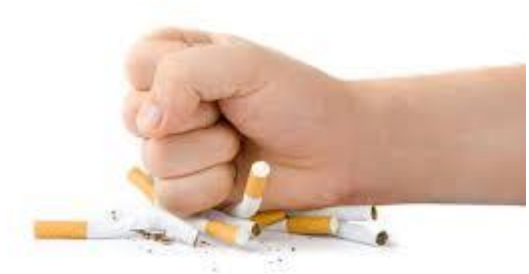

Supplement: Supplementary file 1 [file healthcare-11-00886-s001.zip › healthcare-2273552_supplementarymaterial_information_coronarydisease_patient.pdf]
