# Supplementary material for: Effectiveness of Transition Care Intervention Targeted to High-Risk Patients to Reduce Readmissions: Study Protocol for the TARGET-READ Multicenter Randomized-Controlled Trial
Source: Healthcare (Basel). 2023 Mar 18;11(6):886. doi: 10.3390/healthcare11060886 (PMC10048511; doi:10.3390/healthcare11060886)

[Logo of the recruiting center]

Information sheet to the patient
DIABETES

What is diabetes?
Diabetes is a condition where the sugar level (blood sugar) in the blood is too high due to a lack of insulin production or the body's resistance to insulin. Insulin is a hormone that regulates blood sugar levels and delivers sugar to the various organs. Diabetes is most often a silent disease, with complications arising after several years. They are related to a generalized attack of the arteries which become blocked and thus decrease the perfusion of the organs. This leads to angina pectoris or even heart attacks, strokes, loss of sight, kidney failure, obstruction of the arteries of the legs with pain when walking, and even risk of necrosis and amputation. Nerve damage is also common, with decreased sensitivity in the legs.

What are the goals of treatment?
To avoid these complications, it is important to maintain a blood sugar level as normal as possible.

What does the treatment consist of?
1) Weight loss, healthy eating, and regular exercise
2) Oral medications to lower blood sugar levels, and/or insulin injections if needed.
3) Medication to reduce other cardiovascular risks.
4) Vaccination against pneumococcus and influenza

What are the warning signs?
Diabetes is often asymptomatic and discovered during a check-up.
- When the sugar level is too low (hypoglycemia): dizziness, sweating, palpitations, trembling.
- When the sugar level is too high (hyperglycemia): intense thirst, increased frequency and quantity of urination.

In both cases, this can lead to coma.

[Logo of the recruiting center]

What can I do for my health if I have diabetes?

1) **Check my blood sugar regularly**, and in any case in case of symptoms of hypoglycemia or hyperglycemia.
2) **Always carry fast sugars** (grape sugar, juice, sweetened drink) to take in case of hypoglycemia symptoms. Don't forget to take slow sugars (bread) after the fast sugars if I have a hypoglycemia.
3) In case of frequent blood sugar levels above 10 mmol/L or below 4 mmol/L, consult my doctor to adapt the treatment.
4) **Take my medication as prescribed**. In case of problems with medication, do not stop taking it without contacting my doctor or pharmacist promptly.
5) Follow my diabetic diet.
6) Stop smoking, have a healthy lifestyle, and do regular physical activities.
7) Get a flu shot every year.
8) Make sure I take good care of my feet (hygiene and good shoes).

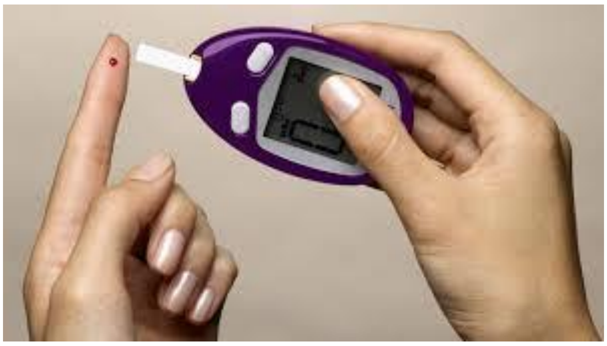

Supplement: Supplementary file 1 [file healthcare-11-00886-s001.zip › healthcare-2273552_supplementarymaterial_information_diabetes_patient.pdf]
