# Supplementary material for: Effectiveness of Transition Care Intervention Targeted to High-Risk Patients to Reduce Readmissions: Study Protocol for the TARGET-READ Multicenter Randomized-Controlled Trial
Source: Healthcare (Basel). 2023 Mar 18;11(6):886. doi: 10.3390/healthcare11060886 (PMC10048511; doi:10.3390/healthcare11060886)
Supplement: Supplementary file 1 [file healthcare-11-00886-s001.zip › healthcare-2273552_supplementarymaterial_information_digestivebleeding_patient.pdf]

Information sheet to the patient
DIGESTIVE BLEEDING

What is a digestive bleed?

It is bleeding from the stomach or intestine. It is most often caused by an ulcer or irritation of the stomach. Some medications increase the risk of digestive bleeding, for example aspirin, corticosteroids, or anticoagulants (Sintrom, Marcoumar, Eliquis, Xarelto). These medications, if prescribed, are still very important. A digestive bleeding manifests itself either by vomiting fresh blood or digested blood (like coffee grounds), or by blood in the stool. Sometimes the blood is not visible and only fatigue is felt due to blood loss.

What are the goals of treatment?

The main goals of treatment are to stop the bleeding, reduce the risk of serious complications or recurrence.
What does the treatment usually consist of?
Endoscopic intervention, i.e. using a thin tube equipped with a camera to locate the source of bleeding and if necessary stop it.
Medication to protect against stomach acid.

When should I consult a doctor?

A digestive bleeding can be silent. Contact your doctor promptly when you have any of the following symptoms:
Presence of traces of blood in vomit or stool.
Fatigue, weakness, or dizziness.
Abdominal pain.
Stools that are coal-black and very foul-smelling.

Contact 144 if you experience:

Fainting or loss of consciousness.
Vomiting blood or "coffee grounds".
Large amount of fresh blood in the stool.

[Logo of the recruiting center]

What can I do for my health if I have digestive bleeding?

1) Check my stool when I go to the bathroom.
2) Avoid medications that can cause ulcers, such as anti-inflammatory drugs, unless my doctor allows it.
3) See my doctor regularly, but also as soon as blood appears in my stool or vomit.
4) Take my medication as prescribed. In case of problems related to medication, do not stop taking it without contacting my doctor or pharmacist promptly.
