# Supplementary material for: Effectiveness of Transition Care Intervention Targeted to High-Risk Patients to Reduce Readmissions: Study Protocol for the TARGET-READ Multicenter Randomized-Controlled Trial
Source: Healthcare (Basel). 2023 Mar 18;11(6):886. doi: 10.3390/healthcare11060886 (PMC10048511; doi:10.3390/healthcare11060886)
Supplement: Supplementary file 1 [file healthcare-11-00886-s001.zip › healthcare-2273552_supplementarymaterial_information_heartfailure_patient.pdf]

[Logo of the recruiting center]

Information sheet to the patient
HEART FAILURE

What is heart failure?
It is a condition where the heart no longer pumps blood sufficiently. As a result, the various parts of the body no longer receive enough oxygen and energy and water accumulates in the body. The most common symptoms are difficulty breathing, fatigue and swollen legs.

What are the goals of the treatment?
The main goals of treatment are to reduce symptoms, slow the progression of the disease, reduce the frequency of decompensation and the risk of death, and improve quality of life.

What does the treatment usually consist of?
Medications to lower blood pressure and protect the heart.
Medications to remove excess water from the legs and lungs
Medication to treat other diseases that can lead to heart failure, such as diabetes, vascular problems.

When should I see a doctor?
Contact your doctor when you have any of the following symptoms:
Swollen legs, weight gain, increased difficulty breathing, or fatigue on exertion, lack of appetite.

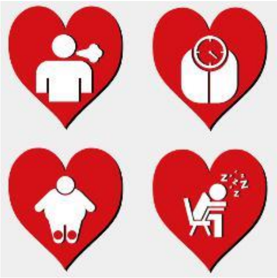

Contact 144 in case of:
- Sudden difficulty breathing.
- Chest pain.
- Faintness or loss of consciousness.
- Palpitations or rapid pulse.
- Faintness, loss of consciousness

What do I need to watch out for or monitor?
Weigh myself once a day under similar conditions (every morning before breakfast), keep track of my weight and consult my doctor if I gain weight (more than 2 kg).

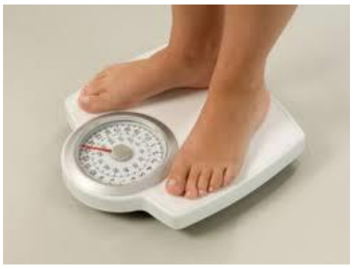

What can I do for my health if I have heart failure?

1) Consult my doctor regularly, and in any case as soon as the symptoms of heart failure appear or worsen.
2) Take my medication as prescribed. In case of problems related to medication, do not stop taking it without contacting my doctor or pharmacist as soon as possible.
3) Monitor my weight, and if it increases, consult my doctor.
4) Get a flu shot at the end of each year and a pneumococcal vaccine.
5) Stop smoking, have a healthy lifestyle, avoid eating salty meals.
