# Supplementary material for: Effectiveness of Transition Care Intervention Targeted to High-Risk Patients to Reduce Readmissions: Study Protocol for the TARGET-READ Multicenter Randomized-Controlled Trial
Source: Healthcare (Basel). 2023 Mar 18;11(6):886. doi: 10.3390/healthcare11060886 (PMC10048511; doi:10.3390/healthcare11060886)
Supplement: Supplementary file 1 [file healthcare-11-00886-s001.zip › healthcare-2273552_supplementarymaterial_information_interventiongroup_familyphysician.pdf]

[Logo of the recruiting center]

Information sheet – family physician

Participation at the TARGET-READ study

Dear Doctor, dear colleague,

Following her/his hospitalization, your patient ____________________ has been assessed as being at high risk of re-hospitalization within 30 days.

What

This notice is part of the Target-READ study funded by the Swiss National Science Foundation to test an intervention to reduce the risk of readmission in high-risk patients.

Why

If you receive this notice, it means that your patient has agreed to take part in the study and has been randomized to the intervention group. Based on the estimation using the validated HOSPITAL score, your patient is estimated to be at high risk for rehospitalization within 30 days.

How

The study does not interfere at any way with your management, and it is up to you to judge what may be useful for your patient to do to reduce his or her risk of rehospitalization.

Our intervention with your patient primarily includes:

- Setting up a follow-up appointment at your office within 7 days.

- Education of the patient on their illnesses and the importance of their treatment.

- A telephone call at D3 and D14 to your patient is made by a nurse. Only advice on medication compliance and lifestyle is given. If necessary, the patient is invited to contact you directly.

Contact

For more information, please visit www.target-read.org or contact us:

**Study nurse**	**Physician locally responsible for the study at your hospital**	**Physician responsible for the study**
[name]<br>[address]<br>[email]<br>[phone number]	[name]<br>[address]<br>[email]<br>[phone number]	Prof. Dr. med. Jacques Donzé<br>[address]<br>[email]<br>[phone number]
