# Supplementary material for: Effectiveness of Transition Care Intervention Targeted to High-Risk Patients to Reduce Readmissions: Study Protocol for the TARGET-READ Multicenter Randomized-Controlled Trial
Source: Healthcare (Basel). 2023 Mar 18;11(6):886. doi: 10.3390/healthcare11060886 (PMC10048511; doi:10.3390/healthcare11060886)
Supplement: Supplementary file 1 [file healthcare-11-00886-s001.zip › healthcare-2273552_supplementarymaterial_information_interventiongroup_patient.pdf]

[Logo of the recruiting center]

Information sheet – intervention group

Your participation at the TARGET-READ study

Dear Sir/Madam,

Thank you for participating in the Target-READ study, which focuses on reducing the risk of re-hospitalization for patients. **You have been selected to be part of the intervention group**. You will receive a visit from our nurse before your discharge to review some information with you. Your family doctor will soon receive a letter from the hospital with information about your hospital stay. **As soon as you are discharged from the hospital, your attending physician will be your health care provider and you should refer to him or her if you have any health problems.**

To help you in your follow-up, **we have already arranged an appointment with your doctor Dr.** ______________________

for the date of: _________________________ at _____h_____

Phone number: ____________________

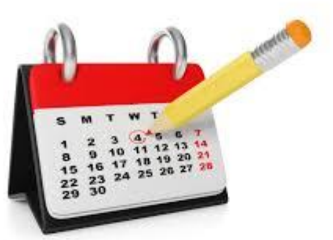

Don't forget to go! Prompt follow-up after discharge from the hospital is essential to avoid further health problems.

A nurse will contact you by phone at 3 days and 2 weeks after your discharge to make sure you are doing well.

In addition, someone will contact you briefly within 1 month to ask you some questions, including whether you had to return to the hospital (**call from the [recruiting hospital] [phone number]**).

We thank you in advance for your very valuable collaboration.

The entire study team wishes you a safe return home and a speedy recovery.

On behalf of the team, Professor Jacques Donzé

<u>Contact</u>

If you have any question, please contact us:

**Study nurse**	**Physician locally responsible for the study at your hospital**	**Physician responsible for the study**
[name]<br>[address]<br>[email]<br>[phone number]	[name]<br>[address]<br>[email]<br>[phone number]	Prof. Dr. med. Jacques Donzé<br>[address]<br>[email]<br>[phone number]
