# Supplementary material for: Effectiveness of Transition Care Intervention Targeted to High-Risk Patients to Reduce Readmissions: Study Protocol for the TARGET-READ Multicenter Randomized-Controlled Trial
Source: Healthcare (Basel). 2023 Mar 18;11(6):886. doi: 10.3390/healthcare11060886 (PMC10048511; doi:10.3390/healthcare11060886)
Supplement: Supplementary file 1 [file healthcare-11-00886-s001.zip › healthcare-2273552_supplementarymaterial_information_peripheralarterialdisease_patient.pdf]

[Logo of the recruiting center]

Information sheet to the patient
PERIPHERAL ARTERIAL DISEASE

What is arterial occlusive disease of the lower limbs?
It is the suffering of a lower limb (from the foot to the buttock) caused by the reduction of oxygen supply, most often due to the obstruction of a blood vessel by atherosclerosis plaques. It is most often manifested by tingling, cramps, or pain in the legs when walking, which disappear at rest. Foot ulcers may also appear.

What are the goals of treatment?
To avoid progression of the disease, to prevent complications.

What does the treatment consist of?
Regular walking to stimulate blood flow in the lower limbs.
Medication to thin the blood such as aspirin.
Stop smoking, take medication for diabetes, cholesterol, and hypertension.
If necessary, allow reperfusion of the affected limb by means of an intervention/operation if necessary: percutaneous angioplasty (dilation of an artery with a balloon), placement of a stent or vascular surgery.

When should I see a doctor?
When I have any of the following symptoms:
- Pain or numbness in one leg when walking, which goes away after a while of rest.
- A toe that turns blue or black.
- Wound that does not heal well.

Contact 144 if you have
- Severe, sharp pain in a leg.
- Pain in the chest.

[Logo of the recruiting center]

What can I do for my health?

1) Consult my doctor regularly, but also as soon as pain or new sores appear or worsen in my legs.
2) Take my medication as prescribed. In case of problems related to medication, do not stop taking it without contacting my doctor or pharmacist as soon as possible.
3) Have a healthy lifestyle: stop smoking, limit fatty foods, regular physical activity (see information sheet on healthy lifestyle).
