# Supplementary material for: Effectiveness of Transition Care Intervention Targeted to High-Risk Patients to Reduce Readmissions: Study Protocol for the TARGET-READ Multicenter Randomized-Controlled Trial
Source: Healthcare (Basel). 2023 Mar 18;11(6):886. doi: 10.3390/healthcare11060886 (PMC10048511; doi:10.3390/healthcare11060886)
Supplement: Supplementary file 1 [file healthcare-11-00886-s001.zip › healthcare-2273552_supplementarymaterial_information_stroke_patient.pdf]

Information sheet to the patient
STROKE

What is a stroke?
It is the suffering or death of a part of the brain caused by the absence of oxygen supply, most often due to the obstruction of a blood vessel, sometimes to an intracranial bleed. It is a medical emergency. It is most often manifested by the sudden appearance of paralysis of a part of the face, difficulty in speaking, weakness of an arm or a leg.

What are the goals of treatment?
To reduce the risk of another stroke.

What is the treatment?
Emergency treatment involves unclogging blocked arteries in the brain with either medication or surgery.
Long-term treatment is important to prevent recurrence and may typically include:
Medication to prevent a new artery blockage (e.g., Aspirin).
Medication to lower blood pressure.
Medicine to reduce high cholesterol.
Medication to "thin" the blood (Sintrom, Eliquis, Xarelto) in case of atrial fibrillation.
Reduction of cardiovascular risk factors (stop smoking, regular physical activity).
Sometimes a rehabilitation cure is useful.

When should I see a doctor?
Consult your doctor regularly to check your blood pressure and generally to ensure that your cardiovascular risk factors are under control.

Contact 144 immediately in case of
- Sudden onset of paralysis in any part of the face
- Difficulty speaking
- Weakness of an arm or leg

[Logo of the recruiting center]

What can I do to prevent another stroke?

1) Immediately call 144 as soon as symptoms appear, even if the stroke only lasts a few minutes. This can be a warning signal for a more severe stroke!
2) Take my medication as prescribed. If there are any problems with the medication, do not discontinue the medication without promptly contacting my doctor or pharmacist.
3) Follow the recommendations for a healthy lifestyle (see general information), stop smoking.
