# Supplementary material for: Effectiveness of Transition Care Intervention Targeted to High-Risk Patients to Reduce Readmissions: Study Protocol for the TARGET-READ Multicenter Randomized-Controlled Trial
Source: Healthcare (Basel). 2023 Mar 18;11(6):886. doi: 10.3390/healthcare11060886 (PMC10048511; doi:10.3390/healthcare11060886)
Supplement: Supplementary file 1 [file healthcare-11-00886-s001.zip › healthcare-2273552_supplementarymaterial_information_thromboembolicdisease_patient.pdf]

[Logo of the recruiting center]

Information sheet to the patient
VENOUS THROMBOEMBOLIC DISEASE:
PULMONARY EMBOLISM/VENOUS THROMBOSIS

What is venous thromboembolic disease (VTE)?
It includes deep vein thrombosis (DVT) and pulmonary embolism (PE). DVT is the obstruction by a clot of a vein, most often in the legs, which manifests itself by a swollen and painful leg. PE is the obstruction of an artery in the lungs by a blood clot (most often originating in the legs, therefore from a DVT), which manifests itself by difficulty in breathing. VTE is a medical emergency.

What are the goals of treatment?
To dissolve the clot, prevent complications and avoid a recurrence.

What does the treatment usually consist of?
The first step is to allow the blood vessel to reperfuse. The duration of treatment depends on the risk of recurrence.
Medication to “thin” the blood (anticoagulant such as Sintrom, Xarelto, Eliquis).
Treatment of risk factors.

When should I see a doctor?
Contact your doctor when you experience any of the following symptoms:
Swollen legs, difficulty breathing, fatigue on exertion.

Contact 144 if you have:
- Sudden difficulty breathing.
- Chest pain.
- Faintness or loss of consciousness.
- Digestive bleeding.

What can I do to avoid another thrombosis/embolism?

1) Take my medication strictly as prescribed and without interruption.
2) Consult my doctor if I am planning a long trip.
